# ﻿New records and a new species of the subgenus Antocha (Antocha) Osten Sacken, 1860 (Diptera, Limoniidae) from Northwest China

**DOI:** 10.3897/zookeys.1256.135717

**Published:** 2025-10-20

**Authors:** Xue Qiao, Xue Wang, Ding Yang, Jinlong Ren

**Affiliations:** 1 Key Laboratory of the Pest Monitoring and Safety Control on the Crop and Forest, College of Agronomy, Xinjiang Agricultural University, Urumqi 830052, China Xinjiang Agricultural University Urumqi China; 2 Department of Entomology, China Agricultural University, Beijing 100193, China China Agricultural University Beijing China

**Keywords:** Key, Shaanxi, taxonomy, Xinjiang

## Abstract

Only a single species of the subgenus Antocha (Antocha)Osten Sacken (1860) was previously known to occur in Northwest China. In this study, four additional species are added to the regional fauna: A. (A.) bimaculata**sp. nov.** is described and illustrated as new species, while A. (A.) chonsaniana Podenas, 2015, and A. (A.) turkestanica de Meijere, 1921, which are reported from China for the first time, are illustrated in detail. Additionally, A. (A.) bifida Alexander, 1924, is recorded for the first time recorded in Northwest China. Furthermore, a key to the species of the subgenus Antocha (Antocha) in Northwest China is presented.

## ﻿Introduction

The subgenus Antocha (Antocha) was established by Osten Sacken 1860 with the type species *Antocha saxicola* Osten Sacken, 1859. A total of 117 species are recognized (Oosterbroek 2025), among these, 51 taxa are found in the Palaearctic Region, seven taxa in the Nearctic Region, 1 taxon in the Neotropical Region, and 68 taxa in the Oriental Region (Oosterbroek 2025). To date, 34 species of Antocha (Antocha) subgenus are known in China, but only one species has been recorded in Northwest China, indicating that this region remains poorly studied for this subgenus. Based on this observation, we conducted field trapping to further investigate and enhance our understanding of the subgenus A. (Antocha). This subgenus is characterized by the following features: body size small to medium-sized; prescutum and presutural scutum usually with three or four stripes, though sometimes faint or even absent; wing with anal angle at nearly a right-angle; crossvein m-cu located before the fork of *M*, sometimes connected to the fork of *M*; male gonocoxites each with two pairs of elongate gonostyli at the apices, inner gonostylus fleshy, sometimes inflated apically; a pair of parameres on both sides of the aedeagus, sometimes inner branched; aedeagus rod-shaped, curved ventrally, sometimes with a bifid apex; female ovipositor with a cercus apically slightly raised upwards and distinctly narrowed (Podenas 2015; Lv et al. 2023).

Northwest China encompasses the following six provinces (or autonomous regions): Xinjiang, Shaanxi, Ningxia, Gansu, Qinghai, and the western part of Inner Mongolia. Until recently, only one species of A. (Antocha) was known to occur in this area: A. (A.) nebulipennis Alexander, 1931. In this study, we describe a new species, A. (A.) bimaculata sp. nov., and provide detailed a description and illustrations. Additionally, we report the first Chinese records of A. (A.) chonsaniana Podenas, 2015 and A. (A.) turkestanica de Meijere, 1921, and provide updated a description and illustrations of A. (A.) bifida Alexander, 1924, which is recorded from Northwest China for the first time. Furthermore, we provide a key to the species of Antocha (Antocha) known from this region of China.

## ﻿Materials and methods

The specimens were collected in Xinjiang, Qinghai, and Shaanxi, China, during July and August 2016 to 2024. The specimens were studied using a Shunyu SZN45 stereomicroscope. Photographs were taken with a Nikon D750 camera equipped with a Canon MP-E 65 mm macro lens (for body and wings), and a Nikon ECLIPSE Ni upright microscope (for hypopygium and ovipositor). Image stacks were created using Adobe Photoshop 2023 (Adobe Systems Ltd). Descriptions were verified using specimens immersed in 75% ethyl alcohol (C_2_H_5_OH). Male genitalia were examined after maceration of the apical portion of the abdomen in hot 10% NaOH for 8–15 min. Studied specimens are deposited in the collections of the Entomological Museum, China Agricultural University (**CAU**), Beijing and Xinjiang Agricultural University (**XJAU**), Xinjiang, China.

The morphological terminology follows Cumming and Wood (2017) and de Jong (2017) for wing venation. The term “inner branch of paramere” is adopted from Kato and Tachi (2019). Species distribution data are based on Oosterbroek (2025). The following abbreviations are used in the figures: aed = aedeagus, app = apical part of paramere, bp = base of paramere, cerc = cercus, goncx = gonocoxite, hyp vlv = hypogynial valve, i gonst = inner gonostylus, ib = interbase, ibp = inner branch of paramere, o gonst = outer gonostylus, pm = paramere, tg 9 = tergite 9, tg 10 = tergite 10.

### ﻿Taxonomy

**Checklist of Antocha (Antocha) crane flies from Northwest China**

A. (A.) bifida Alexander, 1924

A. (A.) bimaculata sp. nov.

A. (A.) chonsaniana Podenas, 2015

A. (A.) nebulipennis Alexander, 1931

A. (A.) turkestanica de Meijere, 1921

### ﻿Key to species (males) of Antocha (Antocha) from Northwest China

**Table:** 

1	Body yellow; prescutum and presutural scutum without stripes (Markevičiūtė 2019: 121, fig. 8)	**Antocha (Antocha) nebulipennis**
–	Body brown or dark brown; prescutum and presutural scutum with stripes	**2**
2	Prescutum and presutural scutum with three stripes (Figs 1c, 5c)	**3**
–	Prescutum and presutural scutum with four stripes (Figs 3c, 7c)	**4**
3	Crossvein m-cu before the fork of *M*, distance approximately half its own length (Fig. 1d); posterior margin of tergite 9 with two widely separated extensions (Fig. 2a); outer gonostylus apically bifid (Fig. 2a)	**Antocha (Antocha) bifida**
–	Crossvein m-cu before the fork of *M*, distance approximately one-quarter its own length (Fig. 5d); posterior margin of tergite 9 without extensions (Fig. 6a); outer gonostylus apically blunt (Fig. 6a)	**Antocha (Antocha) chonsaniana**
4	Aedeagus with apex bifid (Figs 7f, 8a); posterolateral angle of tergite 9 slightly extended and rounded (Fig. 8a); outer gonostylus distinctly curved and apex slightly bifid (Fig. 8a)	**Antocha (Antocha) turkestanica**
–	Aedeagus with apex not bifid (Figs 3e, 4a); posterolateral angle of tergite 9 not extended (Fig. 4a); outer gonostylus slightly curved and apex not bifid (Fig. 4a)	**Antocha (Antocha) bimaculata sp. nov.**

#### ﻿ Antocha (Antocha) bifida

Taxon classificationAnimaliaDipteraLimoniidae

Alexander

B6E52D22-FC64-5F25-9FBB-B639E4892775

Figs 1, 2

Antocha bifida Alexander, 1924: 564; Antocha pallida Lackschewitz 1964: 716 (synonym).

##### Material examined.

China – Xinjiang U.A.R. • 1 ♂; Burqin, Hemu; 1046 m a.s.l.; 48.4301°N, 87.5705°E; 21 Jul. 2016; J.L. Ren leg.; Cau • 1 ♂, 9 ♀♀; Burqin, Hemu, Saibeikezhan; 1098 m a.s.l.; 48.5700°N, 87.4300°E; 22 Jul. 2016; J.L. Ren leg.; light trap leg.; CAU. – Shaanxi Prov. • 1 ♂, 2 ♀♀; Zhouzhi, Houzhenzhi; 1297 m a.s.l.; 33.8400°N, 107.8300°E; 9 Jul. 2017; X.L. Li leg.; CAU • 1 ♂, 1 ♀; Yangxian, Huaishuguan; 1297 m a.s.l.; 33.2398°N, 107.7100°E; 12 Jul. 2017; Y.P. Chen leg.; Cau • 20 ♂♂, 23 ♀♀; Yangxian, Yishuizhen, Mujiacun; 690 m a.s.l.; 33.2990°N, 107.4360°E; 2 Aug. 2017; X.L. Chen leg.; Cau • 3 ♂♂, 15 ♀♀; Yangxian, Yaopingxiang, Yaoshuba; 600 m a.s.l.; 33.3969°N, 107.3542°E; 3 Aug. 2017; X.L. Chen leg.; light trap leg.; Cau • 11 ♂♂, 18 ♀♀; Yangxian, Huayangzhen; 1110 m a.s.l.; 33.6098°N, 107.3969°E; 4 Aug. 2017; X.L. Chen leg.; light trap leg.; Cau • 3 ♂♂, 4 ♀♀; Yangxian, Huayangzhen, Yonggou; 1315 m a.s.l.; 33.6198°N, 107.3952°E; 5 Aug. 2017; X.L. Chen leg.; light trap leg.; Cau • 20 ♂♂, 23 ♀♀; Yangxian, Yishuizhen, Mujiacun; 911 m a.s.l.; 33.3512°N, 107.4299°E; 8 Aug. 2017; X.L. Chen leg.; Cau • 10 ♂♂, 20 ♀♀; Yangxian, Huayangzhen, Yantou; 1206 m a.s.l.; 33.6188°N, 107.3928°E; 8 Aug. 2017; X.L. Chen leg.; Cau • 30 ♂♂, 43 ♀♀; Yangxian, Maopingzhen, Shaojiagou; 911 m a.s.l.; 33.4288°N, 107.6789°E; 8 Aug. 2017; X.L. Chen leg.; CAU. – Gansu Prov. • 18 ♂♂, 14 ♀♀; Tianshui, Zhangjialinchang; 1339 m a.s.l.; 34.6800°N, 106.3300°E; 13 Jul. 2017; J.L. Zhou leg.; Cau • 5 ♂♂, 3 ♀♀; Tianshui, Zhangjialinchang; 1339 m a.s.l.; 34.6800°N, 106.3300°E; 13 Jul. 2017; Qilemoge leg.; Cau – Qinghai Prov. • 4 ♂♂; Menyuan, Sigou; 2405 m a.s.l. 37.1159°N, 102.3499°E; 18 Jul. 2023; X. Wang leg.; XJAU.

##### Diagnosis.

Body yellow. Head brown. Antenna short, reaching pronotum if bent backward. Prescutum and presutural scutum with four yellow stripes, central stripes divided 2/3 posteriorly. Scutellum with cloud-shaped brown markings, large stripe in middle, two markings at upper-anterior corners. Wing with pale brown cloud-shaped pattern around most veins; stigma grayish-brown; crossvein m-cu nearly connected to *M* fork. Outer gonostylus goose-head-shaped at apex, mesal part slightly narrower than remaining; inner branch of paramere elongated, with apex acute. Aedeagus tongue-shaped.

##### Redescription.

**Male.** Body length 4.48–5.80 mm, wing length 4.43–6.12 mm, antenna length 0.71–0.83 mm (*N* = 128).

**Figure 1.**
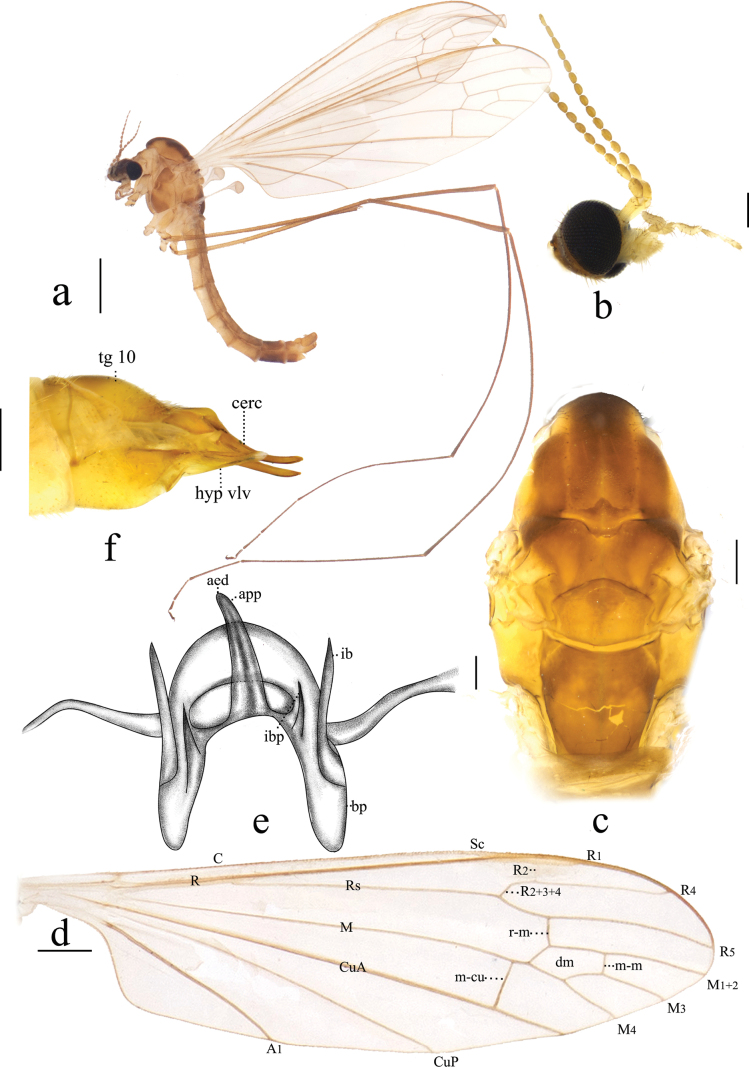
Antocha (Antocha) bifida. A. Habitus of male, lateral view; B. Male head, lateral view; C. Male thorax, dorsal view; D. Right wing; E. Aedeagal complex, dorsal view; F. Female ovipositor, lateral view. Scale bars: 1.0 mm (A); 0.5 mm (D); 0.2 mm (B, C, F); 0.05 mm (E).

***Head*** (Fig. 1a, b). Mostly brown. Vertex brown. Antenna pale brown, 16-segmented, reaching base of pronotum if bent backward; scape cylindrical; pedicel narrower at base, with brown distal margin; flagellomeres oval, distal segments elongated, cylindrical, with erect sparse setae (Fig. 1a, b).

***Thorax*** (Fig. 1a, c). Mostly brown. Pronotum brown, with dark-brown apex. Prescutum and presutural scutum yellow, with four indistinct brown stripes; median stripes partially divided posteriorly for approximately 2/3 of their length, with a narrow yellow seam between them. Postsutural scutum brown, scutal lobes each with blunt square pale spots at lower posterior corners. Scutellum brown with dark-brown lateral margin. Mediotergite brown, dark-brown marking at upper-anterior corners, with bright-yellow stripes on the upper side of these markings (Fig. 1c). Legs with fore-coxae brown; mid-coxae and hind-coxae lighter; trochanters yellow, with brown margins; femora pale brown, slightly swollen distally; tibiae pale brown, with brown apices; tarsi brown, pale brown at base of first tarsi (Fig. 3a). Wing whitish subhyaline; stigma pale brown; veins pale brown, *R_1_*, *CuA_1_* and *A_2_* dark brown; crossvein m-cu before *M* fork (Fig. 1d). Halter pale brown, subhyaline; knob pale brown (Fig. 1a).

***Abdomen*** (Fig. 1a). Mostly brown. Sternites 7–8 with dark-brown stripe in middle. Abdominal setae brownish.

**Figure 2.**
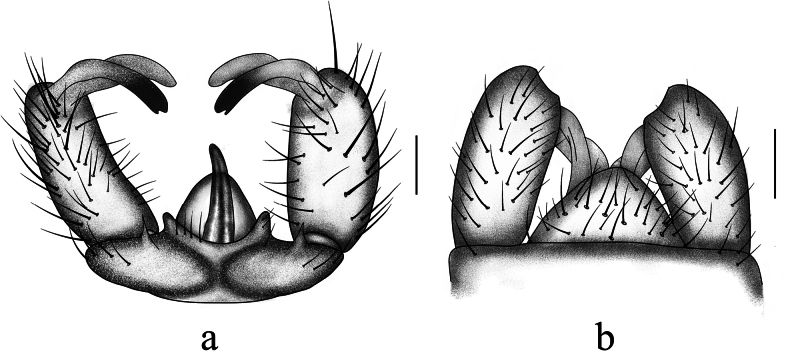
Antocha (Antocha) bifida. A. Male hypopygium, dorsal view; B. Male hypopygium, ventral view. Scale bar: 0.1 mm.

***Hypopygium*** (Figs 1e, 2). Mostly brown. Tergite 9 with horned projections and shallow depression at mid-posterior margin (Fig. 2a). Gonocoxite elongated and cylindrical (Fig. 2). Outer gonostylus sclerotized, blunt at apex; small, shallow depression located approximately 2/3 along anterior end. Inner gonostylus fleshy, slightly broader than outer gonostylus, with sparse yellow setae; apex blunt. Aedeagal complex with parameres fused apically, forming flattened arch, base of paramere elongate and slightly curved, extending laterally with a gradual taper at apex. (Figs 1g, 2a). Inner branch of paramere in the shape of elongated spike, blackened at apex. Interbase a narrow, elongated, apically acute lobe. Aedeagus tongue-shaped.

**Female.** Body length 5.82–6.34 mm, wing length 5.89–6.71 mm, antenna length 0.60–0.83 mm (*N* = 175). Resembles male in head and wing.

***Ovipositor*** (Fig. 1f). Tergite 10 yellow, with brown spot. Cercus narrow, apex slightly raised and tapering. Hypogynial valve straight, yellow, reaching about 1/2 of cercus.

##### Elevation range in China.

Adults were collected at altitudes ranging from 600 m to 2400 m.

##### Period of activity.

Adults were collected in July and August.

##### Distribution.

China (Gansu, Guangdong, Qinghai, Shaanxi, Sichuan, Taiwan, Xinjiang), Japan, Kazakhstan, Mongolia, North Korea, Philippines, Russia, South Korea.

#### ﻿ Antocha (Antocha) bimaculata sp. nov.

Taxon classificationAnimaliaDipteraLimoniidae

9C6909EB-8F01-5F36-83FF-A6AAE1BD1188

https://zoobank.org/606BA3C5-AE17-42E0-8D32-F11F5D5CAE8F

Figs 3, 4

##### Type material.

**Holotype**: China – Qinghai Prov. • ♂; Menyuan, Laohugou; 3029 m a.s.l.; 37.4478°N, 101.5622°E; 26 Jul. 2023; X. Wang leg.; light trap; XJAU. **Paratypes**: China – Qinghai Prov. • 1 ♂; Menyuan, Sigou; 2405 m a.s.l.; 37.1159°N, 102.3499°E; 18 Jul. 2023; X. Wang leg.; light trap; XJAU • 1 ♂; Menyuan, Sanchakou; 2709 m a.s.l.; 37.2078°N, 102.1880°E; 21 Jul. 2023; X. Wang leg.; XJAU • 2 ♂♂; Menyuan, Heiyegou; 2863 m a.s.l.; 37.2527°N, 102.3424°E; 23 Jul. 2023; X. Wang leg.; XJAU • 2 ♂♂; Menyuan, National Highway 569; 2580 m a.s.l.; 37.2334°N, 102.0173°E; 24 Jul. 2023; X. Wang leg.; XJAU.

##### Diagnosis.

Body brown to dark brown. Antenna long, reaching wing base if bent backward. Prescutum and presutural scutum dark brown, with four dark stripes. Mediotergite dark brown, about twice the length of scutellum, with oval, brown spot at posterior margin. Wing without stigma; crossvein m-cu before *M* fork, distance about 1/5 its own length. Tergite 9 with posterior margin flat, shallow depression at middle. Outer gonostylus slightly curved, distal half blackened, apex blunt. Aedeagal complex with a large sheath at both ends, formed by the apical part of the parameres.

##### Description.

**Male**. Body length 6.66–7.41 mm, wing length 7.61–8.31 mm, antenna length 1.13–1.34 mm (*N* = 6).

**Figure 3.**
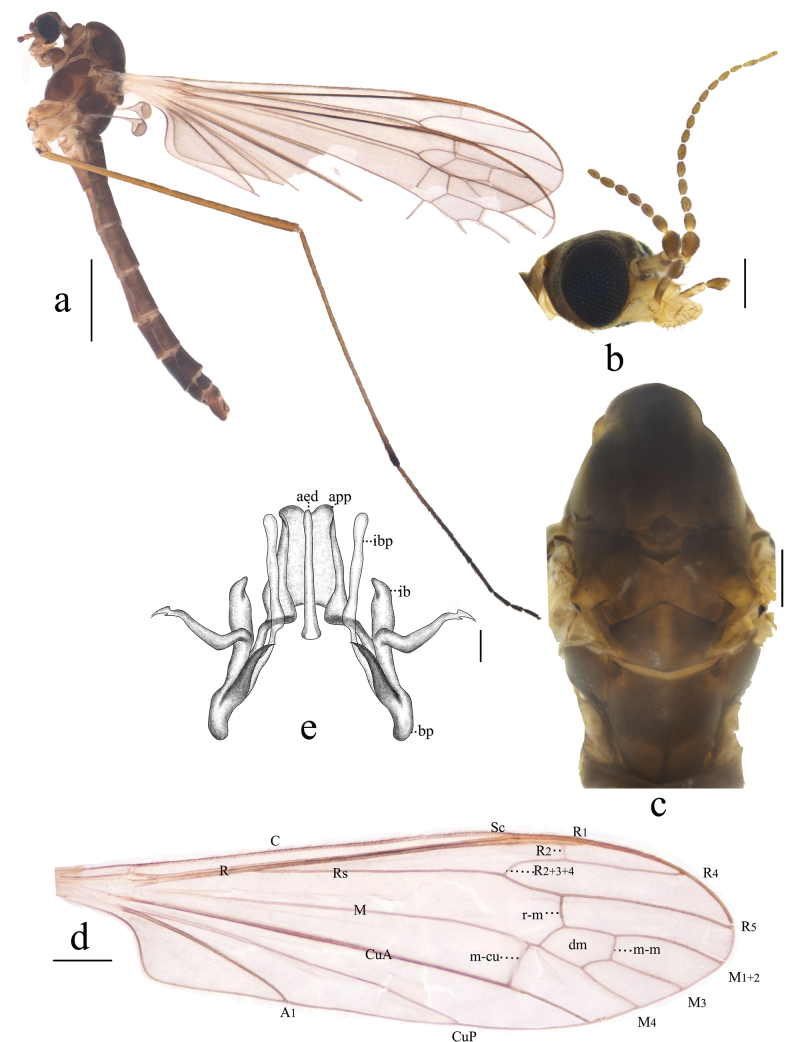
Antocha (Antocha) bimaculata sp. nov. A. Habitus of male, lateral view; B. Male head, lateral view; C. Male thorax, dorsal view; D. Right wing; E. Aedeagal complex, dorsal view. Scale bars: 1.0 mm (A); 0.5 mm (D); 0.2 mm (B, C); 0.05 mm (E).

***Head*** (Fig. 3a, b). Mostly dark brown. Vertex with sparse, brown setae along eye margin. Antenna brown, 16-segmented, reaching wing base if bent backward; scape cylindrical, enlarged; flagellomeres dark brown, terminal four segments pale brown (Fig. 3a, b).

***Thorax*** (Fig. 3a, c). Mostly dark brown. Pronotum dark brown. Prescutum and presutural scutum dark brown, with four indistinct dark stripes. Postsutural scutum dark brown, middle area yellowish brown. Scutellum dark brown with yellowish-brown band at middle. Mediotergite long, dark brown, twice as long as scutellum, posterior margin with two oval, brown spots (Fig. 3c). Legs with fore-coxae dark brown; mid-coxae and hind-coxae lighter, pale-brown markings; trochanters pale brown, with dark-brown margins; femora brown, slightly swollen distally; tibiae brown, with dark-brown apices; tarsi dark brown (Fig. 3a). Wing gray subhyaline, without stigma; veins generally brown, *R_1_* and *Sc* with apices pale yellow; crossvein m-cu before *M* fork, distance about 1/5 its length; cell dm approximately twice as long as wide (Fig. 3d). Halter with stem gray, knob brown in distal half.

***Abdomen*** (Fig. 3a). Mainly dark brown. Tergites 1–6 grayish brown, tergites 7–8 dark brown. Sternites 1–6 pale brown, sternites 7–8 dark brown. Abdominal setae brownish.

**Figure 4.**
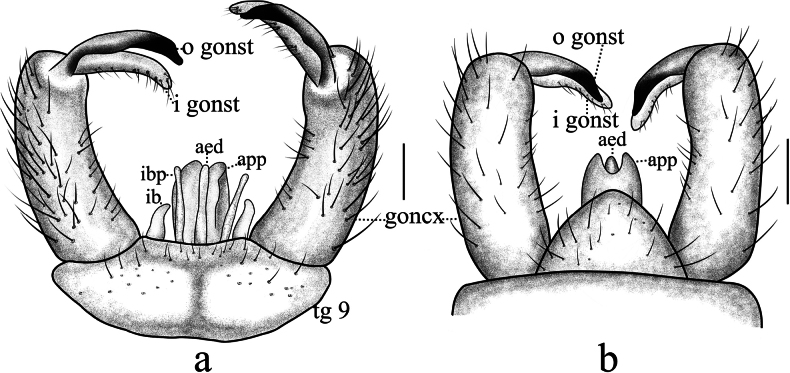
Antocha (Antocha) bimaculata sp. nov. A. Male hypopygium, dorsal view; B. Male hypopygium, ventral view. Scale bar: 0.1 mm.

***Hypopygium*** (Figs 3e, 4). Mostly brown. Tergite 9 dark brown, wider than long; posterior margin flat, strongly sclerotized, densely covered with setae, with shallow depression at middle (Fig. 4a). Gonocoxite nearly cylindrical, densely covered by long, brown setae (Fig. 4). Outer gonostylus slightly curved, distal half blackened, apex blunt. Inner gonostylus slightly curved, with short, sparse setae. Aedeagal complex with parameres exhibiting a distinct apical excavation, large sheath at both ends, base of paramere elongated; inner branch of paramere elongated, membranuous, apically rounded; interbase short, wider than inner branch of paramere and aedeagus, apically blunt. Aedeagus rod-shaped (Figs 3e, 4a).

**Female.** Unknown.

##### Elevation range in China.

Adults were collected at altitudes ranging from 2000 m to 3000 m.

##### Period of activity.

Adults were collected only in late July.

##### Distribution.

China (Qinghai: Menyuan).

##### Etymology.

The specific name (from Latin *bimaculatus* (adj., meaning “two spots”) refers to the two spots at the posterior margin of mediotergite.

##### Remarks.

This new species closely resembles A. (A.) styx Alexander, 1930 in body coloration, the morphology of male head, wings and hypopygium. However, it can be distinguished from the latter based on the following characteristics: in A. (A.) bimaculata sp. nov., the flagellomeres are ellipsoidal, with the last two segments being elongated; the prescutum and presutural scutum are entirely dark brown, featuring four indistinct dark stripes; the mediotergite is dark brown, with two oval brown spots on the posterior margin; and the outer gonostylus without emargination on its inner margin. In A. (A.) styx, the flagellomeres decrease in size towards the apex; the prescutum and presutural scutum are dark brown with lighter areas laterally and three confluent blackish-brown stripes; the mediotergite is blackish brown, with indistinct brownish spots on both sides; and the outer gonostylus has a small emargination on its inner margin (Podenas and Yang 2015).

#### ﻿ Antocha (Antocha) chonsaniana

Taxon classificationAnimaliaDipteraLimoniidae

Podenas

7BD29BB5-2555-5B51-A3C6-FBD2B45E41D6

Figs 5, 6

Antocha (Antocha) chonsaniana Podenas, 2015: 3.

##### Material examined.

China – Gansu Prov. • 25 ♂♂; Tianshui, Zhangjialinchang; 1339 m a.s.l.; 34.6800°N, 106.3300°E; 13 Jul. 2017, Qilemoge leg.; CAU • 22 ♂♂, 2 ♀♀; Tianshui, Zhangjialinchang; 1339 m a.s.l.; 34.6800°N, 106.3300°E; 13 Jul. 2017, J.L. Zhou leg.; Cau. – Shaanxi Prov. • 2 ♂♂, 1 ♀; Yangxian, Maopingzhen, Shaojiagou; 911 m a.s.l.; 33.4288°N, 107.6789°E; 8 Aug. 2017, X.L. Chen leg.; CAU.

##### Diagnosis.

Body brown. Antenna short, reaching base of prescutum if bent backward. Prescutum and presutural scutum with three darker brown stripes on brown background. Wing grayish brown; stigma slightly darker, barely noticeable; crossvein m-cu before *M* fork, distance about 1/4 its length. Tergite 9 with posterior margin having shallow depression at middle; outer gonostylus strongly sclerotised, wide at base, narrowed to blunt apex. Aedeagus sword-shaped, blunt at apex. Inner branch of paramere elongated, blackened and blunt at apex. Interbase a narrow, elongated, apically acute lobe. Ovipositor with long, narrow cercus, apex raised, tapering; hypogynial valvae long, straight, reaching half cercus.

##### Description.

**Male**. Body length 4.39–4.87 mm, wing length 4.54–4.79 mm, antenna length 0.86–1.16 mm (*N* = 49).

**Figure 5.**
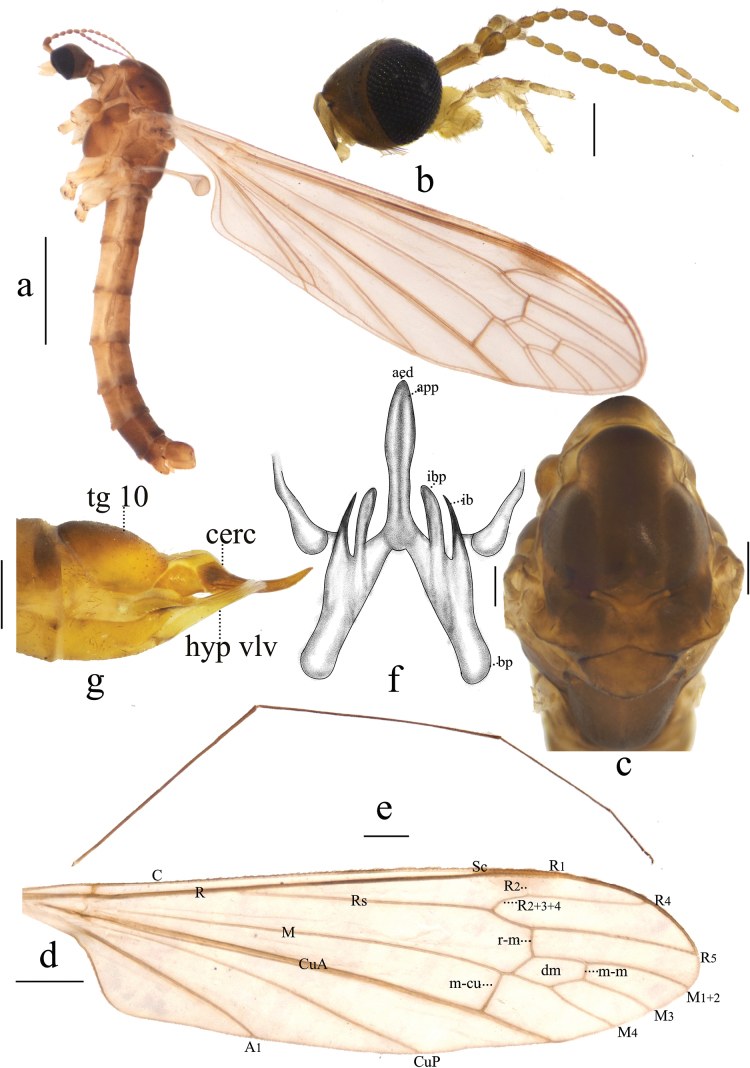
Antocha (Antocha) chonsaniana. A. Habitus of male, lateral view; B. Male head, lateral view; C. Male thorax, dorsal view; D. Right wing; E. Male leg, lateral view; F. Aedeagal complex, dorsal view; G. Female ovipositor, lateral view. Scale bars: 1.0 mm (A, E); 0.5 mm (D); 0.2 mm (B, C, G); 0.05 mm (F).

***Head*** (Fig. 5a, b). Mostly pale brown. Vertex brown. Antenna pale brown, 16-segmented, reaching base if bent backward; scape cylindrical; pedicel narrower at base, distal margin brown; flagellomeres oval, distal segments elongated, cylindrical, with erect, sparse setae (Fig. 5a, b).

***Thorax*** (Fig. 5a, c). Mostly brown. Pronotum brown, with large dark brown spot in middle. Prescutum and presutural scutum brown, with three dark-brown stripes. Postsutural scutum brown, scutal lobes each with blunt, square, dark spots on anterior middle. Scutellum brown, with black lateral margin. Mediotergite brown, dark-brown marking at upper-anterior corners, with pale-brown stripes on the upper side of these markings (Fig. 5c). Legs with fore-coxae pale brown; mid-coxae and hind-coxae lighter; trochanters pale brown with brown margins; femora pale brown, slightly swollen distally; tibiae brown, brown apices; tarsi brown, pale brown at base of first tarsi (Fig. 5a, e). Wing grayish brown; stigma slightly darker, barely noticeable; *R_1_*, *CuA_1_* and *A_2_* brown; crossvein m-cu before *M* fork, distance about 1/4 its length (Fig. 5d). Halter pale brown, subhyaline; knob pale brown (Fig. 5a).

***Abdomen*** (Fig. 5a). Mostly pale brown. Sternites 7–8 with brown stripe in middle. Abdominal setae brownish.

**Figure 6.**
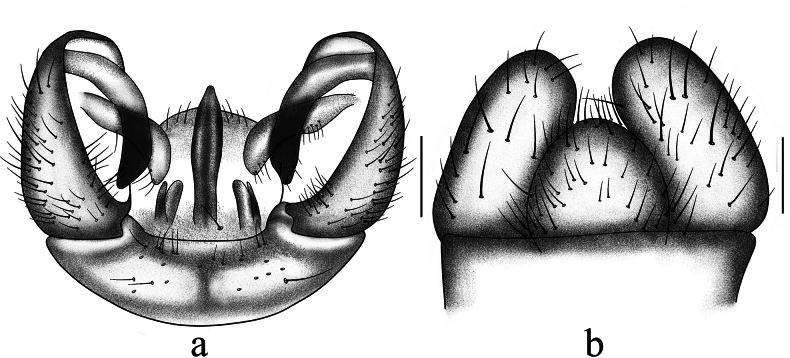
Antocha (Antocha) chonsaniana. A. Male hypopygium, dorsal view; B. Male hypopygium, ventral view. Scale bar: 0.1 mm.

***Hypopygium*** (Figs 5f, 6). Mostly brown. Tergite 9 with dark-brown stripes in middle, shallow depression at mid-posterior margin (Fig. 6a). Gonocoxite cylindrical, inner margin concave (Fig. 6). Outer gonostylus strongly sclerotized, wide at base, narrowed to blunt apex, bent inward. Inner gonostylus fleshy, slightly broader than outer gonostylus, covered with sparse, yellow setae; apex blunt. Aedeagal complex with parameres fused apically, base of paramere elongated, extending laterally with a gradual taper at apex (Figs 5f, 6a). Inner branch of paramere elongated, blackened and blunt at apex. Interbase a narrow, elongated, apically acute lobe. Aedeagus sword-shaped, blunt at apex.

**Female.** Body length 5.29–6.05 mm, wing length 5.37–5.61 mm, antenna length 0.84–1.24 mm (*N* = 3). Resembles male in head and wing.

***Ovipositor*** (Fig. 5g). Tergite 10 yellow, with brown spot. Cercus long, narrow, apex slightly raised and tapering. Hypogynial valve, straight, yellow, reaching about 1/2 of cercus.

##### Elevation range in China.

Adults were collected at altitudes ranging from 900 m to 1400 m.

##### Period of activity.

Adults were collected in July and August.

##### Distribution.

China (Gansu: Tianshui; Shaanxi: Yangxian), North Korea.

##### Remark.

This is the first report of this species from China.

#### ﻿ Antocha (Antocha) turkestanica

Taxon classificationAnimaliaDipteraLimoniidae

de Meijere

1A5A9BAB-C270-583D-9DAB-79338A3847E7

Figs 7, 8, 9

Antocha turkestanica de Meijere, 1921: 107; Antocha libitina Alexander,1960: 159 (synonym); Taphrophila afghana Nielsen, 1962: 167 (synonym).

##### Material examined.

China – Xinjiang U.A.R. • 7 ♂♂; Gongliu, Kuerdening; 1140 m a.s.l.; 43.2549°N, 82.8250°E; 26 Jul. 2017, J.L. Ren leg.; Cau • 1 ♂; Zhaosu, Qiongbola; 1815 m a.s.l.; 43.4399°N, 81.0098°E; 30 Jul. 2017, B. Zhang leg.; Cau • 10 ♂♂, 3 ♀♀; Wenquan, Heba; 1300 m a.s.l.; 44.9799°N, 81.0354°E; 31 Jul. 2017, J.L. Ren leg.; Cau • 3 ♂♂, 1 ♀; Yumin, 165 Regiment; 902 m a.s.l.; 46.8478°N, 86.7853°E; 2 Aug. 2017, J.L. Ren leg.; Cau • 10 ♂♂; Yumin, Tasi river; 987 m a.s.l.; 48.6602°N, 86.7895°E; 3 Aug. 2017, B. Zhang leg.; Cau • 2 ♂♂; Zhaosu, Balekesu; 1634 m a.s.l.; 43.0478°N, 81.2450°E; 24 Jul. 2024, J.L. Ren leg.; light trap; XJAU • 2 ♂♂, 1 ♀; Tekesi, Tekesi; 2037 m a.s.l.; 42.9149°N, 82.1894°E; 27 Jul. 2024, J.L. Ren leg.; light trap; XJAU • 30 ♂♂, 9 ♀♀; Gongliu, Qiaxisenlin; 1680 m a.s.l.; 43.0582°N, 82.7471°E; 27 Jul. 2024, J.L. Ren leg.; light trap; XJAU • 50 ♂♂, 23 ♀♀; Gongliu, Qiaxisenlin; 2026 m a.s.l.; 43.0590°N, 82.5981°E; 28 Jul. 2024, X. Qiao leg.; XJAU.

##### Diagnosis.

Body dark brown. Antenna long, reaching wing base if bent backward. Prescutum and presutural scutum with four dark-brown stripes, central stripes divided 3/4 posteriorly; mediotergite dark brown, without grain. Wing pale gray, without stigma; vein *CuA* brown, wider and darker than other veins; crossvein m-cu almost connected to *M* fork. Tergite 9 with Y-shaped depression at middle, divided into two lobes, posterolateral margin with round projection; outer gonostylus strongly curved, base yellow, distal 2/3 blackened, apex slightly bifid. Aedeagus apically bifid; inner branch of paramere dark brown, base darker; interbase a narrow, apically blunt lobe.

##### Description.

**Male**. Body length 6.11–6.38 mm, wing length 6.57–6.89 mm, antenna length 0.83–0.99 mm (*N* = 115).

**Figure 7.**
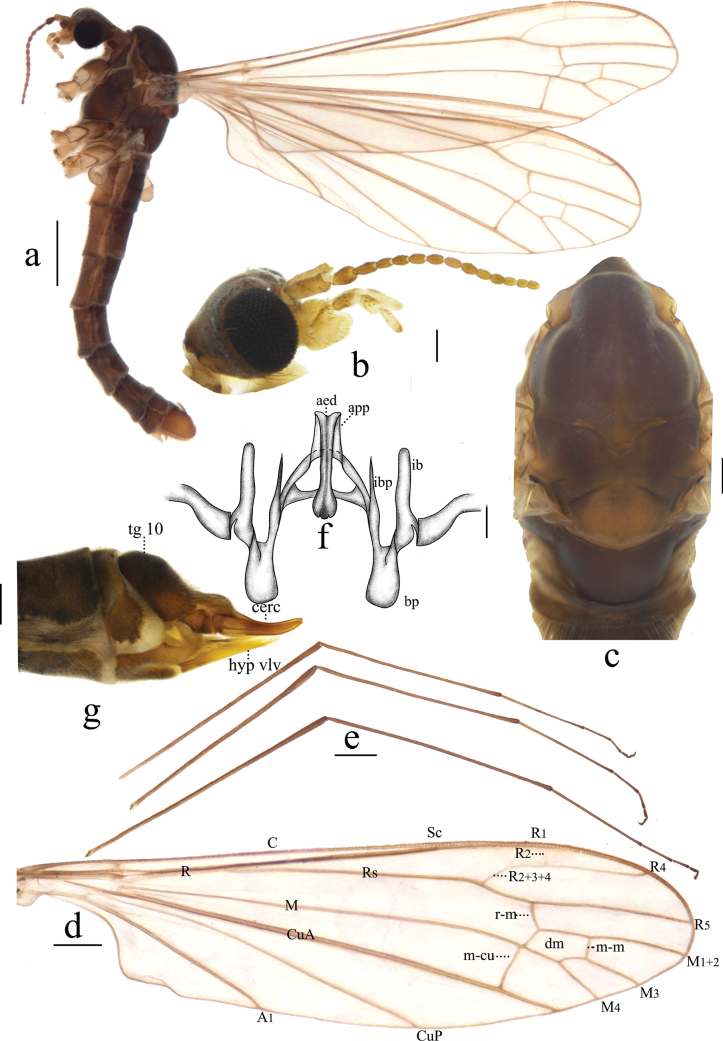
Antocha (Antocha) turkestanica. A. Habitus of male, lateral view; B. Male head, lateral view; C. Male thorax, dorsal view; D. Right wing; E. Male leg, lateral view; F. Aedeagal complex, dorsal view; G. Female ovipositor, lateral view. Scale bars: 1.0 mm (A, E); 0.5 mm (D); 0.2 mm (B, C, G); 0.05 mm (F).

***Head*** (Fig. 7a, b). Mostly brown. Vertex dark brown. Antenna light brown, 16-segmented, reaching wing base if bent backward; scape cylindrical; pedicel narrower at base, brown distal margin; flagellomeres oval, distal segments elongated cylindrical, with erect sparse setae and pale tips (Fig. 7a, b).

***Thorax*** (Fig. 7a, c). Mostly brown. Pronotum brown, large dark-brown spot in middle. Prescutum and presutural scutum brown, with four dark brown stripes; central stripes divided 3/4 posteriorly, with pale-brown seam; mediotergite dark brown, without grain. Postsutural scutum brown, middle and posterior margin lighter in color. Scutellum brown with dark-brown lateral margin. Mediotergite dark brown (Fig. 7c). Legs with fore-coxae dark brown; mid-coxae and hind-coxae lighter; trochanters pale brown; femora pale brown, slightly swollen distally; tibiae pale brown, brown apices; tarsi brown, pale brown at base of first tarsi (Fig. 7a, e). Wing pale gray, without stigma; vein *CuA* brown, wider and darker than other veins; crossvein m-cu almost connected to *M* fork (Fig. 7d). Halter pale brown, subhyaline; knob pale brown (Fig. 7a).

***Abdomen*** (Fig. 7a). Mostly brown. Sternites 7–8 with dark brown stripe in middle. Abdominal setae black.

**Figure 8.**
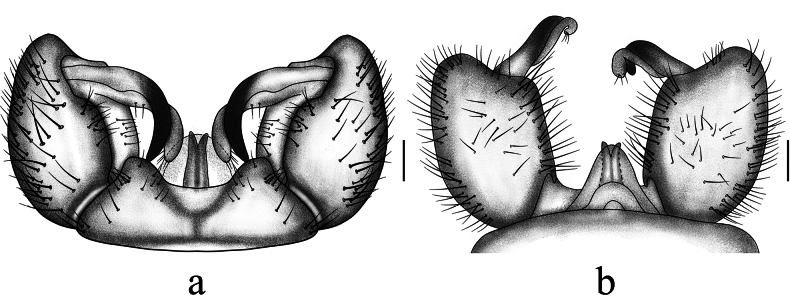
Antocha (Antocha) turkestanica. A. Male hypopygium, dorsal view; B. Male hypopygium, ventral view. Scale bar: 0.1 mm.

***Hypopygium*** (Figs 6, 7e). Mostly brown. Tergite 9 with Y-shaped depression at middle, divided into two lobes, posterior margin with round projection on each side (Fig. 8a). Gonocoxite cylindrical, inner margin concave (Fig. 8). Outer gonostylus strongly curved, base yellow, distal 2/3 blackened, apex slightly bifid. Inner gonostylus fleshy, slightly broader than outer gonostylus, with sparse, yellow setae, blunt apex. Aedeagal complex with parameres fused apically, forming flattened arch, base of paramere clover-shaped, with a gradual taper at apex (Figs 7f, 8a). Inner branch of paramere gradually tapering from the middle to the apex, dark brown, base darker. Interbase a narrow, elongated, apically acute lobe. Aedeagus bifid at apex.

**Female.** Body length 6.86–7.89 mm, wing length 7.94–8.61 mm, antenna length 1.24–1.33 mm (*N* = 37). Resembles male in head and wing.

***Ovipositor*** (Fig. 7g). Tergite 10 yellow, with brown spot. Cercus long, narrow, apex slightly raised and tapering. Hypogynial valvae, straight, yellow, reaching about 2/3 of cercus.

##### Elevation range in China.

Adults were collected at altitudes ranging from 900 m to 2100 m.

##### Period of activity.

Adults were collected in July and August.

##### Biology.

**Figure 9.**
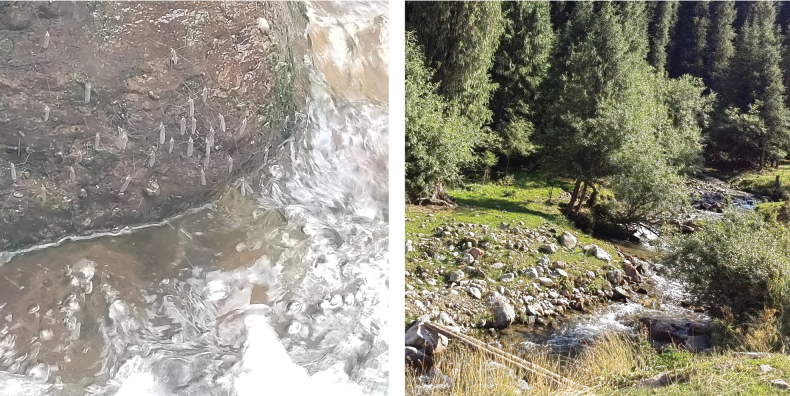
The habitat of Antocha (Antocha) turkestanica, Qiaxisenlin, Gongliu, Xinjiang, China on 28 July 2024.

The adults of this species exhibit phototaxis and gregarious behavior (Fig. 9). They typically inhabit areas near flowing streams and rest on rocks adjacent to the water surface during the daytime.

##### Distribution.

Afghanistan, China (Xinjiang: Yumin, Gongliu, Tekesi, Wenquan, Yumin, Zhaosu), Kazakhstan, Tajikistan, Turkmenistan.

##### Remark.

This is the first report of this species from China.

## Supplementary Material

XML Treatment for Antocha (Antocha) bifida

XML Treatment for Antocha (Antocha) bimaculata

XML Treatment for Antocha (Antocha) chonsaniana

XML Treatment for Antocha (Antocha) turkestanica

## ﻿Additional information

### Use of AI

No use of AI was reported.

### Author ORCIDs

Xue Qiao https://orcid.org/0009-0004-5070-891X

Xue Wang https://orcid.org/0009-0001-4605-1810

Ding Yang https://orcid.org/0000-0002-7685-3478

Jinlong Ren https://orcid.org/0000-0001-5948-1724

### Data availability

All of the data that support the findings of this study are available in the main text.
